# Robotic Gastrointestinal Surgery: State of the Art and Future Perspectives

**DOI:** 10.3390/jpm13030568

**Published:** 2023-03-22

**Authors:** Marco Milone, Paolo Pietro Bianchi

**Affiliations:** 1 Department of Clinical Medicine and Surgery, University of Naples “Federico II”, 80131 Naples, Italy; 2 Department of Health Science, University of Milan, 20142 Milano, Italy

Since its inception, robotic surgery has made incredible progress and has undergone significant development in an extremely short period of time. In the field of minimally invasive surgery, robotic platforms could potentially be used to realize improvements for both the patient and surgeon. Several barriers of laparoscopic surgery could be overcome through the introduction of 3D vision, stable and magnified images, EndoWrist instruments, physiologic tremor filtering, and motion scaling. Minimally invasive surgery is constantly evolving so as to allow surgeons to achieve essential goals in terms of survival and functionality; robotic platforms could play a key role in obtaining these goals. For this reason, it is necessary to evaluate the oncological and functional outcomes of robotic surgery.

Regarding surgical oncology, robotic surgery may offer several benefits through precise visualization and dissection along the embryological planes. One example of its advantageous application is rectal cancer surgery, especially in the case of male narrow pelvis and bulking tumors [1]. Even in esophageal cancer surgery, the robotic approach appears to be slightly superior to laparoscopic surgery, resulting in less postoperative pneumonia and higher numbers of harvested nodes [2].

If the results of robotic surgery, in oncological terms, are encouraging, the same can be said for gastrointestinal functional disorders. Even if, on the one hand, robotic surgery proved to be non-inferior to laparoscopic surgery in the treatment of functional esophageal disorders [3], on the other hand, it showed better postoperative outcomes in the treatment of pelvic floor disorders, such as lower complication rates and shorter lengths of hospital stay [4].

Robotic surgery has also begun to play an important role in endoluminal surgery with the evolution of systems for transanal surgery that allow for the execution of highly complex procedures, such as RTaTME, for low-lying rectal cancer [5].

Although, to date, there are no specific indications for the use of robotic surgery compared to laparoscopic surgery, the former’s lower unplanned conversion rate has been amply demonstrated in the literature. Therefore, one of the targets of this surgery could be patients with known or suspected abdominal adhesions [6].

This Special Issue concerns the application of robotic surgery in the context of gastrointestinal surgery and its safety and efficacy in the performance of various procedures, even those of high complexity. Increased costs, poor availability and dedicated training are still barriers which prevent the widespread adoption of this system. In the near future, emerging robotic platforms will lead to major competition and consequent reductions in costs, encouraging the use of this platform and raising its potential as a standard surgery for many procedures.
